# Supplementary material for: The Characterization of Three Novel Insect-Specific Viruses Discovered in the Bean Bug, Riptortus pedestris
Source: Viruses. 2022 Nov 11;14(11):2500. doi: 10.3390/v14112500 (PMC9696879; doi:10.3390/v14112500)
Supplement: Supplementary file 1 [file viruses-14-02500-s001.zip › viruses-1982120-supplementary.pdf]

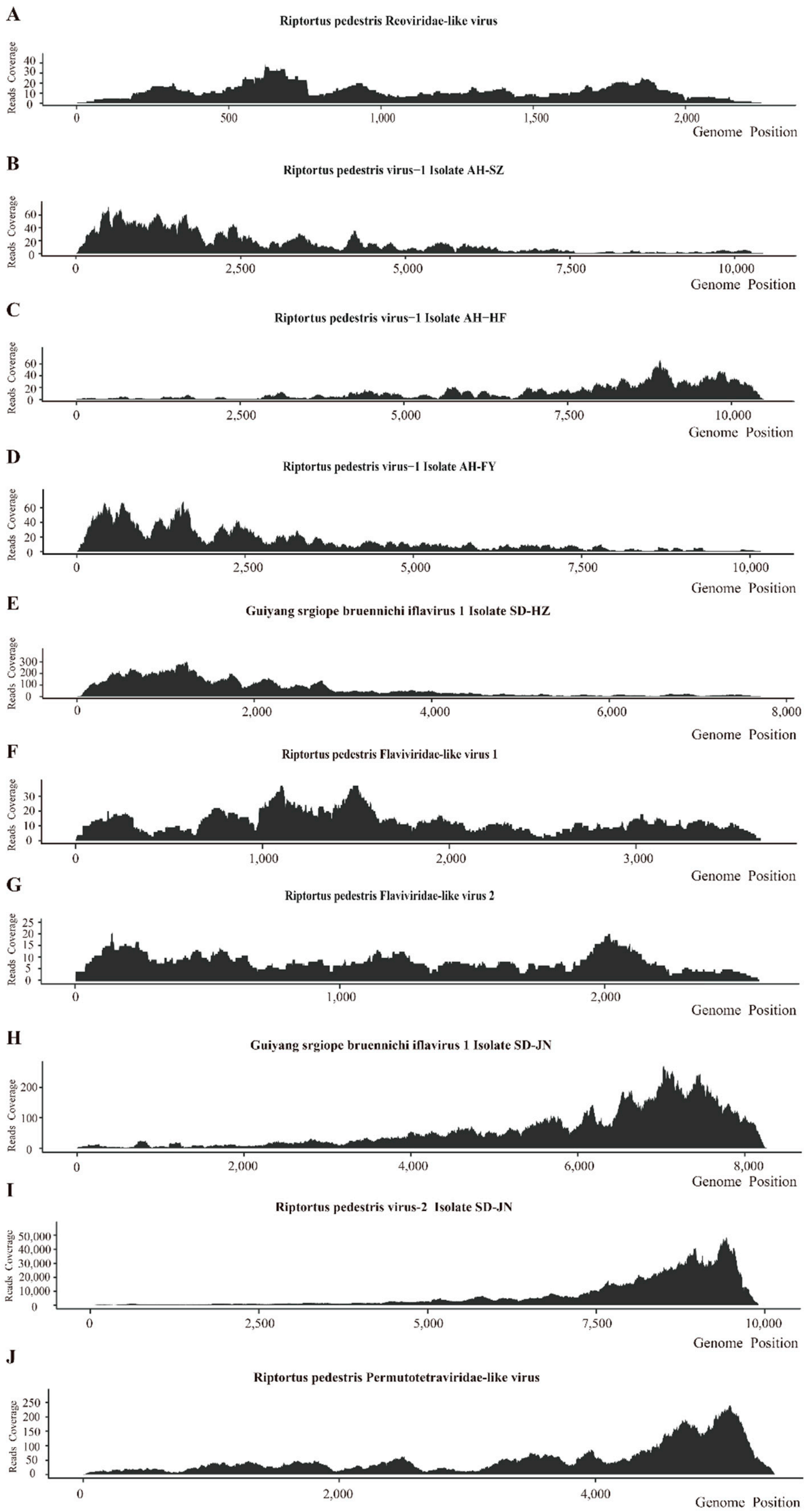

Figure S1. Transcriptome raw read coverage of insect-specific viruses identified in *R. pedestri*

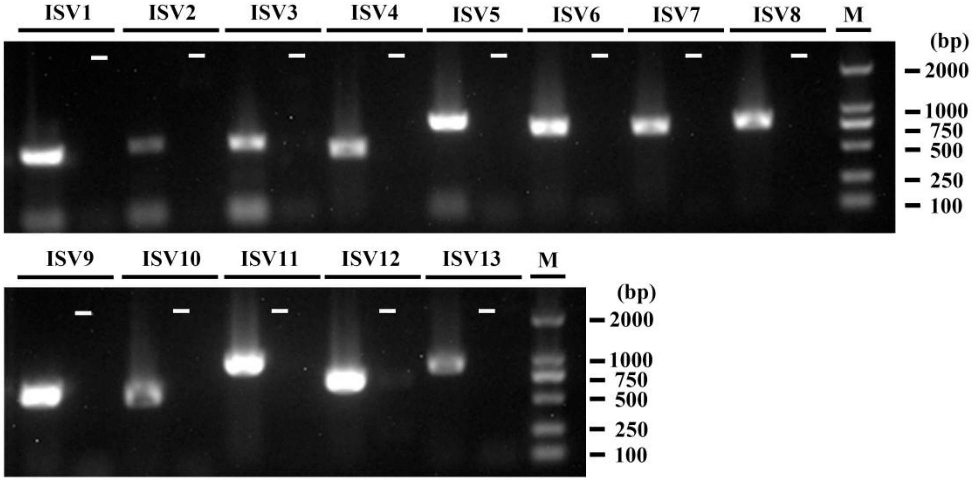

Figure S2. The ISVs in *R. pedestris* were detected by RT-PCR

ISV1, RiPV-1 Isolate AH-SZ; ISV2, RiPV-1 Isolate AH-HF; ISV3, RiPV-1 Isolate AH-FY; ISV4, Guiyang srgiope bruennichi iflavirus 1 Isolate SD-HZ; ISV5, Guiyang srgiope bruennichi iflavirus 1 Isolate SD-JN; ISV6, RiPV-2 Isolate SD-JN; ISV7, RpRLv; ISV8, RpALv1; ISV9, RpALv2; ISV10, RpPLv; ISV11, RiALv; ISV12, RpFLv1; ISV13, RpFLv2; -, negative control, the template is double distilled water.

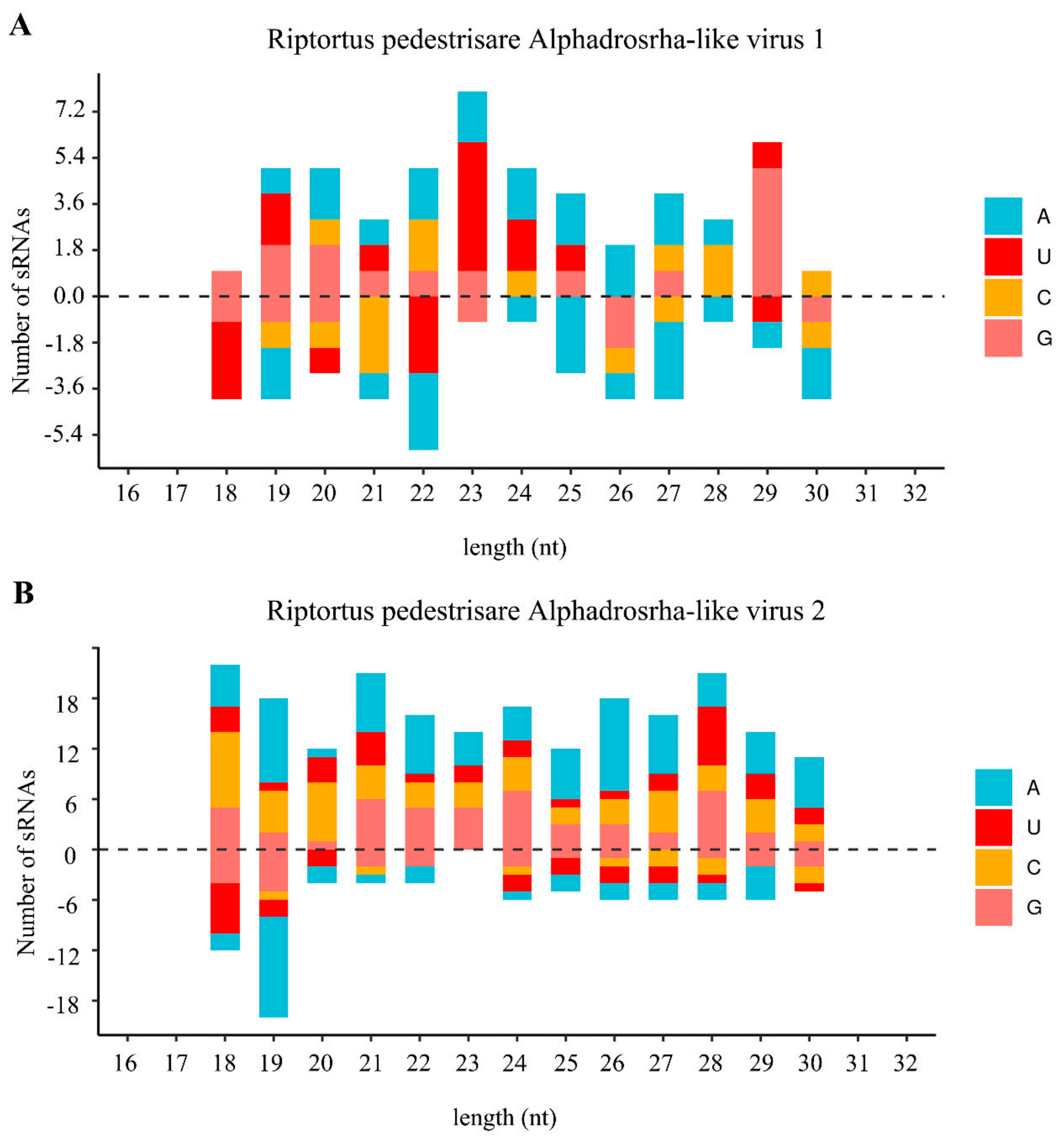

Figure S3. Profile of virus-derived small interfering RNAs (vsiRNAs)

vsiRNAs derived from Riptortus pedestris alphadrosrha-like virus 1 (RpALv1) (A) and Riptortus pedestris alphadrosrha-like virus 2 (RpALv2) (B)**.** Positive and negative values indicate that the vsiRNA was derived from the positive-and negative-sense strand. Color coding shows different nucleotides.

Supplementary Table S1. Primer sequences used for RT-PCR detection

ISV	Primer name	Primer sequence
Riptortus pedestris virus-1 Isolate AH-SZ	RiPV-1-1-F	CTAAGGCTGTCGCTTGGTTC
	RiPV-1-1-R	TCCGGACAGAAAGCAGAGTT
Riptortus pedestris virus-1 Isolate AH-HF	RiPV-1-2-F	CCCTAGCAGTGCTCTTGGTC
Riptortus pedestris virus-1 Isolate AH-FY	RiPV-1-2-R	CACCTACACTCTGGCCCAAT
Guiyang srgiope bruennichi iflavirus 1 Isolate SD-HZ	Srbiv-1-F	AATACGCGCCCAACTGTAAC
	Srbiv-1-R	GCACCAGGATCTCGTTCAAT
Guiyang srgiope bruennichi iflavirus 1 Isolate SD-JN	Srbiv-2-F	AGATGCACGATTGTCTGCTG
	Srbiv-2-R	CCAACGAGGAGCTACAAAGC
Riptortus pedestris virus-2 Isolate SD-JN	RiPV-2-F	GCAGGTGAAGGTCGGTGTAT
	RiPV-2-R	CACAGTTGGTGGAACAATGC
Riptortus pedestris Reoviridae-like virus (RpRLv)	RpRLv-F	GCGCGTTTTCTTAGTTGGTC
	RpRLv-R	GTCTCCCGCAATCGTTCTAC
* Riptortus pedestris Alphadrosrha-like virus 1 (RpALv1)	RpALv1-F	TGACCCTCCACAGTCATTCA
	RpALv1-R	TTTCCAGGGTGTCAACTTCC
* Riptortus pedestrisare Alphadrosrha-like virus 2 (RpALv2)	RpALv2-F	GCTCTGGTTGATCAGTGCAA
	RpALv2-R	AATAGGGTTGGCCCATTACC
Riptortus pedestris Permutotetraviridae-like virus (RpPLv)	RpPLv-2-F	CCCAATGAGGACCCTGTCTA
	RpPLv-2-R	TGGGACTTTCTGGACCAAAC
* Riptortus pedestris Almendra-like virus (RiALv)	RiALv-F	GGTTCGGCGTTACACAGATT
	RiALv-R	TGTAATGGCCTCGGATAAGG
Riptortus pedestris Flaviviridae-like virus 1 (RpFLv1)	RpFLv1-F	TCATTCCCTTGCCTAACCAC
	RpFLv1-R	GGGACCATCGCTAAATGAGA
Riptortus pedestris Flaviviridae-like virus 2 (RpFLv2)	RpFLv2-F	TAGGTTTTCGCCATGGGTAG
	RpFLv2-R	CCGTTTCGGATTACAGCACT

* The novel insect-specific viruses identified in *R. pedestris.*

Supplementary Table S2. *R. pedestris* datasets used in this study

Library	Run accession number（RNA-seq）	Run accession number（sRNA）	Location	Table base (Gb)	N50	RNA-seq Contigs	RNA-seq reads
AH-A1	SRR21672252	SRR21673402	Suzhou, Anhui, China	2.3	1395	102803	43838042
AH-A2	SRR21672251	SRR21673401	Hefei, Anhui, China	2.8	1790	146704	48782946
AH-A3	SRR21672250	SRR21673400	Fuyang, Anhui, China	2.4	1348	111627	50259312
AH-A4	SRR21672249	SRR21673399	Xuancheng, Anhui, China	1.9	1037	85034	40465772
SD-B1	SRR21672248	SRR21673398	Heze, Shandong, China	2.2	2085	95816	41060716
SD-B2	SRR21672247	SRR21673397	Jining, Shandong, China	1.8	976	97482	42591978
